# Outcome Reporting Variability in Trials of Chinese Medicine for Hyperlipidemia: A Systematic Review for Developing a Core Outcome Set

**DOI:** 10.1155/2021/8822215

**Published:** 2021-06-10

**Authors:** Geng Li, Ruxue Han, Wencong Cao, Zehuai Wen, Xiankun Chen

**Affiliations:** ^1^The Second Affiliated Hospital of Guangzhou University of Chinese Medicine, Guangzhou 510120, Guangdong, China; ^2^Key Unit of Methodology in Clinical Research, Guangdong Provincial Hospital of Chinese Medicine, Guangzhou 510120, Guangdong, China; ^3^Foshan Hospital of Traditional Chinese Medicine, Foshan 528000, Guangdong, China; ^4^Second Clinical Medical College (Second Affiliated Hospital), Guangzhou University of Chinese Medicine, Guangzhou 510405, China; ^5^State Key Laboratory of Dampness Syndrome of Chinese Medicine, The Second Affiliated Hospital of Guangzhou University of Chinese Medicine, Guangzhou 510120, Guangdong, China; ^6^Guangdong Provincial Key Laboratory of Clinical Research on Traditional Chinese Medicine Syndrome, Guangzhou 510120, Guangdong, China; ^7^Department of Global Public Health, Health Systems and Policy, Karolinska Institute, Stockholm 17177, Sweden

## Abstract

**Introduction:**

Hyperlipidemia is an underlying process behind cardiovascular disease. Chinese medicine (CM) may be effective in treating hyperlipidemia, but there is a lack of studies with high methodological quality. A major reason for this is heterogeneity in outcome reporting. Therefore, this study explores the degree of outcome reporting variation in CM trials for hyperlipidemia. It then generates a list of potentially important outcomes for developing a core outcome set (COS).

**Methods:**

A systematic review of literature focusing on studies of CM for hyperlipidemia was conducted. Outcomes were listed verbatim and grouped into 8 domains. Outcome frequency and definition uniformity were analyzed.

**Results:**

3,702 studies and 452 individual outcomes were identified. These outcomes were reported 27,328 times, of which 1.6% were reported as primary outcomes, and 13.3% were defined. The most frequent outcome was total triglyceride, represented in 86.7% of the studies, followed by total cholesterol (86.0%), total effective rate (75.1%), high-density lipoprotein cholesterol (73.2%), and low-density lipoprotein cholesterol (60.5%). However, 43.6% of outcomes were reported only once. The largest outcome domain was “pathological or pathophysiological outcomes,” which included 67.0% of outcomes. Of the “response rate related outcomes” domain, total effective rate was the most frequently reported outcome (*n* = 2,780), and 95.3% of the studies gave a clear definition. However, these definitions were often contradictory. Only 10 papers reported cardiovascular events, 3 of which referred to them as primary outcomes. Moreover, ten patient-reported outcomes were reported in the retrieved literature 19 times in total. The majority of the outcomes did not report measurement instruments (MIs) (269/453, 59.4%). MIs of the surrogate outcomes were reported more frequently.

**Conclusion:**

Outcome reporting in CM trials for hyperlipidemia is inconsistent and ill-defined, creating barriers to data synthesis and comparison. Thus, we propose and are developing a COS for CM trials for hyperlipidemia.

## 1. Introduction

Hyperlipidemia is a key underlying process for cardiovascular diseases [1]. It includes hypercholesterolemia, hypertriglyceridemia, and mixed hyperlipidemia [2]. The prevalence of hyperlipidemia among Chinese adults is 34.0%, and it is higher among males than females (41.9% and 32.5%, respectively) [3]. In the United States, an estimated 12.9% of adults have elevated total cholesterol (TC), and 6.2% have undiagnosed hypercholesterolemia [4]. Although hyperlipidemia is prevalent and has received attention from the medical community, it is often neglected by patients. Because clinical hyperlipidemia symptoms are not obvious, patients unfamiliar with it rarely receive serum lipid examinations until their cardiovascular system has already been damaged and symptoms have presented [5]. The awareness, treatment, and control rate of hyperlipidemia in China are low [6, 7], especially in men, aged <45, and especially in rural and Western areas [8]. Many studies have found that Chinese medicine (CM) provides relief to patients with hyperlipidemia [9–11]. One CM treatment, Xuezhikang, has been recommended by the Joint Committee for Developing Chinese Guidelines on Prevention and Treatment of Dyslipidemia in Adults [2].

While trials of the effectiveness of CM for hyperlipidemia are becoming more common, there remains a lack of studies with high methodological quality. Outcome measure is one of the key issues [12]. Several systematic reviews have reported heterogeneity in outcome reporting in many clinical trials [9–11, 13]. Therefore, it is difficult to synthesize clinical trial results with different outcomes. Developing a core outcome set would improve the quality of outcome reporting.

A core outcome set (COS) represents the minimum that should be measured and reported in all clinical trials for a specific condition to facilitate the comparison and combination of trials while researchers continue to explore other outcomes [14]. The Core Outcome Measures in Effectiveness Trials (COMET) initiative collates and stimulates relevant resources, both applied and methodological, to facilitate the exchange of ideas and information and to foster methodological research in this area [14]. The authors have registered COS development for hyperlipidemia with the COMET initiative [15]. Systematic review is a feasible and efficient approach to identifying and aggregating an inclusive list of outcomes being reported by researchers in a given area [16]. It is also recommended by the COMET initiative [17].

This systematic review is the first stage of developing a core outcome set for hyperlipidemia. It explores the degree of heterogeneity of outcome reporting in CM trials for hyperlipidemia and generates a list of potentially important outcomes which will be scored in a Delphi survey.

## 2. Methods

### 2.1. Protocol and Study Registration

This systematic review is a component of the development of a core outcome set for hyperlipidemia. It was registered on the COMET initiative website (registration number: 983) [15]. The protocol for the development of a COS for hyperlipidemia has recently been published [18]. We performed this systematic review in compliance with the preferred reporting items for systematic reviews and meta-analyses (PRISMA) statement [19].

### 2.2. Eligibility Criteria

#### 2.2.1. Type of Studies

Case reports, case-control studies, cohort studies, randomized controlled trials, or systematic reviews, published in either English or Chinese, were included. Studies published only as conference abstracts for which papers could not be accessed, despite sending requests to their authors, were excluded. This was done because conference abstracts are not representative of comprehensive outcome lists, due to their length limitations.

#### 2.2.2. Types of Participants

Patients with hyperlipidemia were included, including high TC, high total triglyceride (TG), mixed high TC and high TG, high low-density lipoprotein cholesterol (LDL-C), and lowered high-density lipoprotein cholesterol (HDL-C), regardless of sex, age, or race. If a study focused on secondary hyperlipidemia, as opposed to other serious diseases, the full text was read to determine whether or not to include it.

#### 2.2.3. Types of Interventions

Patients treated by CM alone or CM in combination with conventional medicine were included

#### 2.2.4. Types of Outcome Measures

All studies reporting hyperlipidemia outcomes were included

### 2.3. Literature Search

Cochrane Central Register of Controlled Trials (CENTRAL), PubMed, Embase, Wanfang Database, China National Knowledge Infrastructure (CNKI), and Chinese Biomedical Database (CBM) were searched, from inception to November 2020. The search strategy included three parts: hyperlipidemia disease, CM treatment, and study type. Boolean operators were used to combine the three parts. Truncations and wildcards were employed to optimize the search. The search strategy is available in Supplementary Materials S1.

### 2.4. Study Selection

Two independent reviewers (GL and RH) initially assessed eligibility by reading titles and abstracts. Any discrepancies were resolved either through discussion after critical review of the full text or by consulting a third author (XC). Duplicate studies were excluded. Studies that did not report hyperlipidemia outcomes were excluded as well.

### 2.5. Data Collection

Two reviewers (GL and RH) independently extracted the data by reading the full texts. Characteristics of each study were extracted, including title, publishing journal, author(s), year of publication, country, authors' affiliation(s), funding, study type, treatment duration, patient source, and diagnosis criteria. The latter included blood lipids, CM syndrome pattern, type of hyperlipidemia, complications, follow-up duration, number of patients who withdrew, intervention details, names of outcomes and whether they had been specified as primary or secondary outcomes, definition of outcomes, time-point and method of outcome measurement, and adverse events.

To ensure the reliability of the analytical details, constant evaluation was conducted between the two reviewers before data extraction. Any disagreements were resolved by discussion and by consulting a third researcher (XC). Data then were extracted using EpiData 3.1 (EpiData Association, Denmark). In cases when the authors had given us missing data by e-mail or telephone, incomplete data were filled in.

To present and analyze the results, outcomes were grouped into eight domains by two researchers (GL and RH). The results were reviewed by another researcher and discussed by a research team. These included mortality related outcomes, pathological or pathophysiological outcomes, response rate related outcomes, cardiovascular events, symptoms or function related outcomes, adverse events or safety related outcomes, patient-reported outcomes, and resource utilization related outcomes.

### 2.6. Statistical Description and Analysis

Data analysis was performed with SPSS 18.0 (IBM SPSS Inc., Armonk, New York, USA). Definitions with the same outcome were extracted and compared. The number of outcomes in each domain and the number of measurement methods were calculated. The frequency of categorical variables was presented, as well as their means and standard deviations, or medians and interquartile ranges for quantitative data.

## 3. Results

### 3.1. Characteristics of Included Studies

**Figure 1:**
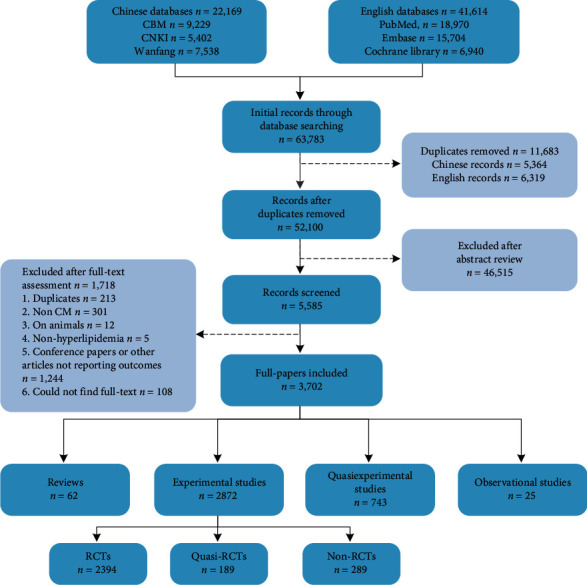
PRISMA diagram of studies searched and included in the systematic review.

The search strategy found 63,783 articles. Finally, 3,702 (3,614 in Chinese and 88 in English) were included after removing duplicates and other ineligible articles (Figure 1).

There were four main types of studies: (1) reviews articles (*n* = 62, 1.7%) including 57 systematic reviews; (2) experimental studies (*n* = 2,872, 77.6%) including randomized controlled trials (RCTs, *n* = 2,394, 64.7%), nonrandomized controlled trials (*n* = 289, 7.8%), and quasirandomized controlled trials (*n* = 189, 5.1%); (3) quasiexperimental studies (*n* = 743, 20.1%); and (4) observational studies (*n* = 25, 0.7%).

The included articles were all published between 1974 and 2020, and 87.0% had been published after 2000. Most of the studies included outpatients and patients with complications. Only 191 (5.4%) studies had funding support. Most of the studies (99.1%) were conducted in China. 1,178 (31.8%) of the studies only included primary hyperlipidemia, and 1,333 (36.0%) included patients with complications.

### 3.2. CM Syndrome Analysis

There were 933 studies reporting CM syndrome patterns, of which 357 included multiple CM patterns. In total, 315 verbatim CM patterns were reported, which were described 1,742 times. After processing and reclassification, 129 CM patterns were identified. The most frequent pattern was *phlegm dampness* (phlegm turbidity) which was reported in 365 studies. It was followed by the *phlegm dampness and blood stasis* pattern (*n* = 277), *qi stagnation and blood stasis* pattern (*n* = 160), *liver-kidney yin deficiency* pattern (*n* = 159), *spleen-kidney yang deficiency* pattern (*n* = 114), and *blood stasis* pattern (*n* = 108). There were 62 syndromes reported only once.

### 3.3. Results on Outcomes

A total of 452 individual recorded outcomes were identified and reported, from which there were 27,328 times (instances). These outcomes were categorized into 5 themes (death, physiological/clinical, adverse events, life impact, and resource use) and 8 domains (mortality related outcomes, pathological or pathophysiological outcomes, response rate related outcomes, cardiovascular events, symptom or function related outcomes, adverse events or safety related outcomes, patient-reported outcomes (PROs), and resource utilization related outcomes). Of note, “physiological/clinical” outcomes were mostly reported by researchers. Details of the five themes and eight domains including their definitions as well as the included outcomes are listed in Table 1.

The ten most frequently reported outcomes among the 3,702 included studies are listed in Table 2. The most frequent outcome was TG, representing 86.7% of the studies (*n* = 3,211), followed by TC in 86.0% of the studies (*n* = 3,185), total effective rate in 75.1% of the studies (*n* = 2,780), HDL-C in 73.2% of the studies (*n* = 2,709), and LDL-C in 60.5% of the studies (*n* = 2,204). Most of the studies neither used them as primary outcomes nor gave clear definitions (Table 2). Note that 197 (43.6%) outcomes were reported only once, and 157 (34.7%) were reported between 2 and 10 times (Supplementary Materials S2).

#### 3.3.1. Mortality Related Outcomes

Mortality related outcomes were reported infrequently, and none reported a detailed definition. There were 2 outcomes relating to morality, all-cause mortality and death, in only 4 studies. One of these studies specified death as the primary outcome, and the other did not.

#### 3.3.2. Pathological or Pathophysiological Outcomes

There were 303 different pathological or pathophysiological outcomes, which were subdivided into 30 subcategories (Table 3). These outcomes were reported 17,036 times, 62.3% of all reported outcomes. Thirty-seven outcomes pertained to blood lipid, the four most common of which were TG, TC, HDL-C, and LDL-C. These four outcomes were reported 11,345 times, accounting for 41.5% of all reported outcomes. Of these, only 310 (2.7%) were reported as primary outcomes, and 259 (2.3%) were defined.

#### 3.3.3. Response Rate Related Outcomes

Twenty-seven individual outcomes measured disease recurrence or progression. Total effective rate was the most frequently reported outcome (*n* = 2,780), although only 18 studies referred to it as a primary outcome. Nevertheless, 2,648 (95.3%) studies defined it. However, based on a categorical assessment (e.g., “markedly improved,” “improved,” “slightly better,” or “no effect”), the definition of total effective rate varied across studies. For example, some studies defined total effective rate as the sum of the “markedly improved” rate and the “improved” rate, while others stated total effective rate as the sum of the “clinical control” (blood lipid returning to normal after intervention) rate, “markedly improved” rate, and “improved” rate. Furthermore, the standard of “improved” also varied. For example, some studies specified that LDL-C that had declined by at least 20% had improved, while others defined improvement as LDL-C below 3.640 mmol/L after treatment.

#### 3.3.4. Cardiovascular Events

10 studies reported cardiovascular events, and 3 of them referred to it as a primary outcome.

#### 3.3.5. Symptom or Function Related Outcomes

This section encompassed 6 subcategories: (1) clinical symptoms (non-CM), (2) CM symptoms or patterns, (3) endothelial function, (4) erectile function, (5) balanced capacity test, and (6) others, with 55 different outcomes. CM syndrome efficacy, CM syndrome score, and clinical signs and symptoms were the three most commonly reported outcomes, reported in 395 (10.7%), 333 (9.0%), and 295 (8.0%) of the studies, with an 85.3%, 42.3%, and 16.6% definition percentage, respectively. Based on reading their definitions, it was discovered that most of these three outcomes were measured by scales. For example, the obstruction of phlegm-dampness pattern grading scale measured the CM score. Tongue manifestation and pulse condition, two important CM symptoms, were reported 54 and 50 times. In addition, nitric oxide (NO) and endothelin were the most commonly reported endothelial function outcomes, reported by 54 and 39 studies, respectively. There were only 10 outcomes reported for erectile function, balanced capacity test, and other functions. Examples included erection quality and static balance with closed eyes.

#### 3.3.6. Adverse Events and Safety Related Outcomes

Three subcategories (adverse events, adverse reactions, and safety outcomes) were considered part of the adverse event or safety domain, with 56 outcomes in total. Adverse events were any untoward medical occurrence in a patient or clinical investigation subject administered a pharmaceutical product which does not necessarily have a causal relationship with this treatment. On the other hand, all noxious and unintended responses to a medicinal product related to any dose should be considered adverse drug reactions [20]. In this paper, adverse events related outcomes only included outcomes with literal meaning of adverse events, such as the number of adverse events and adverse events rates; adverse reactions related outcomes also only included outcomes with literal meaning of adverse reactions, such as the number of adverse reactions and adverse reaction rate. Other safety related outcomes except adverse events and adverse reactions were included in safety outcomes, such as laboratory abnormalities. Liver function, kidney function, routine blood, and urine and stool tests were the most frequently reported outcomes, reported in 1,199 (32.4%), 1,046 (28.3%), 958 (25.9%), 873 (23.6%), and 376 (10.2%) studies, respectively. However, these 5 most common outcomes were rarely defined (the definition incidence rate was below 1%). Other safety related outcomes included hematological or biochemical measures, such as glutamic-pyruvic transaminase (ALT). With regard to adverse events and adverse reaction related outcomes, the most commonly reported outcome was adverse events (reported in 85 studies). The other 4 outcomes (incidence of adverse reactions, adverse reactions, incidence of adverse events, and total incidence of adverse reactions) were reported by 21 papers.

#### 3.3.7. Patient-Reported Outcomes

Ten outcomes were patient-reported. Seven studies reported on quality of life, of which two used SF-36, one used an instrument designed by the researcher, and two used other scales. The rest did not specify the scales used. Additional outcomes were related to activity, health, patient satisfaction, eating behaviors, and mental states or symptoms; all were reported in the individual studies.

#### 3.3.8. Resource Utilization Related Outcomes

Two outcomes related to resource utilization were reported in 3 studies: cost and treatment implementation time.

### 3.4. Outcome Measurement Instrument Results

The measurement instruments (MIs) for most outcomes were not reported (269/453, 59.4%). MIs for surrogate outcomes were reported more frequently; for example, enzymatic analysis and immunoturbidimetry were used for TC measurement. For some of the subjective outcomes, such as quality of life and CM syndrome efficacy, MIs were also reported. For example, CM symptom score scale, CM syndrome rating scale, dyslipidemia syndrome evaluation scale, and *spleen-strengthening and lipid-regulating therapy* questionnaire were used to measure CM syndrome efficacy.

## 4. Discussion

In this systematic review, variation and inconsistencies in the definition and reporting of outcomes were identified. 452 individual outcomes were reported in the included studies, while there was no single outcome reported in all studies. Of those, 43.6% were reported only once, and 34.7% were reported between two and ten times. Most studies did not distinguish primary outcomes from secondary outcomes, nor did they provide explicit definitions of the outcomes measured. Some provided only classification outcomes without detailed outcome measures such as blood lipids, routine blood test, renal function, or carotid ultrasonography. This heterogeneity complicates comparison and synthesis across studies. Thus, the studies' usefulness in promoting clinical practice progress was limited.

A positive finding was that blood lipid outcomes, such as TG, TC, HDL-C, and LDL-C, were reported by most studies. A composite outcome called total effective rate was also extensively reported. However, its definitions varied, inhibiting comparison across studies.

However, cardiovascular events and patient-reported outcomes were rarely reported. One reason may have been that the intervention periods for most of the included studies were not long enough to observe the cardiovascular events. It is also possible that hyperlipidemia is commonplace and usually does not affect patient quality of life [5].

In CM theory, pattern (also called syndrome) is a diagnostic conclusion based on pathological changes in a disease, at a certain stage [21]. A pattern often contains several CM symptoms, such as tongue manifestation or pulse condition. It is important for CM physicians to measure pattern and CM symptom changes when treating patients. Several CM outcomes were reported in this review such as CM symptoms score (primary symptoms and secondary symptoms), CM syndrome efficacy (single or multiple items), tongue manifestation, and pulse condition. However, the definitions and measurement instruments varied. This presents an obstacle to high-quality systematic review. Furthermore, these outcomes were poorly reported—CM syndrome efficacy was the most frequently reported CM outcome, despite only 395 of 3,702 studies reporting it.

Previous work has found heterogeneity in outcome reporting in hyperlipidemia studies. A 2015 systematic review of 35 trials of Zhibituo (a Chinese patented drug) in hyperlipidemia treatment reported 30 different outcomes [13]. A 2011 systematic review of 22 RCTs on Chinese herbal medicines for hypercholesterolemia found 20 individual outcomes, but no trial reported outcomes pertaining to cardiovascular events [11]. The present review covered a wide range of studies. This included not only RCTs, but also observational studies. It identified an exhaustive list of outcomes reported in studies of CM for hyperlipidemia, and it is the first stage of developing a core outcome set for clinical trials of CM for hyperlipidemia.

This review has several limitations, and its results should be interpreted with caution. Firstly, only published studies were included. This may have caused bias. For example, only researchers' and clinicians' viewpoints on outcomes were referenced, while patients and other stakeholders were not considered. However, as the present study is the first stage of developing a core outcome set, we will include several patients and other stakeholders in a future Delphi survey to capture their comments. Secondly, as a literature systematic review, publication lag bias is almost inevitable, which is another limitation of this review. The review compiled the outcomes in the included studies to form the core outcome set experts questionnaire for Delphi survey. In the follow-up survey, we will set up an open item for experts to fill in supplementary suggestions. We believe that invited clinical experts, especially those familiar with hyperlipidemia, will provide their appropriate supplements based on the latest knowledge, such as various clinical practice guidelines. Thirdly, only studies in English and Chinese were included. Thus, outcomes reported in other languages could have been omitted. In consideration of the plethora of studies included, we believe that all important outcomes for hyperlipidemia have been identified.

### 4.1. Implication for Future Research

As the first stage in the process of developing a core outcome set for hyperlipidemia, this review has demonstrated the heterogeneity of outcomes reported in the current literature. It has also generated a list of potentially important outcomes for the next step in hyperlipidemia COS development. In the future, the identified outcome will first be evaluated by the study advisory group, and then COS candidate items will be developed and scored in a Delphi survey. Finally, a consensus meeting will be held with clinicians, patients, and other key stakeholders to finalize items and definitions. A COS for hyperlipidemia will also be developed.

## 5. Conclusion

This systematic review has highlighted the fact that outcome reporting for CM trials of hyperlipidemia has been inconsistent and ill-defined. The results of this review will generate a list of potentially important outcomes for the next step in hyperlipidemia COS development.

## Figures and Tables

**Table 1:** Outcome domains, definitions, number of individual outcome measurements in each domain, and frequency of outcomes reported of each domain.

Theme	Outcome domain	Definition of outcome domain	No. of outcomes	Frequency of outcomes reported
Total			452	27,328
I. Death	1. Mortality related outcomes	Outcomes related to short- and long-term survival/death rates and cause of death	2	4

*II. Physiological/clinical*	2. Pathological or pathophysiological outcomes	Outcomes related to the reporting of blood, or biochemical measures, within standard clinical practice or research	303	17,036
	3. Response rate related outcomes	Measures of disease recurrence or disease progression	23	2,835
	4. Cardiovascular events	Death from coronary heart disease, nonfatal myocardial infarction, fatal or nonfatal ischemic stroke, unstable angina requiring hospitalization, coronary revascularization procedures, peripheral revascularization procedures, heart failure requiring hospitalization, stent thrombosis, and transient ischemic attack [1]	1	10
	5. Symptom or function related outcomes	Signs and symptoms of disease reported by clinicians	55	1,358

III. Adverse events	6. Adverse event or safety related outcomes	Forms of short-term and long-term complications	56	6,063
IV. Life impact	7. Patient-reported outcomes	Outcomes reported by patients themselves	10	19
V. Resource use	8. Resource utilization related outcomes	Economic outcomes related to healthcare	2	3

[1] Sabatine MS, Giugliano RP, Wiviott SD, et al. Efficacy and safety of evolocumab in reducing lipids and cardiovascular events. N Engl J Med, 2015, 372:1500–1509.

**Table 2:** The ten most frequently reported outcomes among the 3,702 included studies.

Outcome name	Domain	No. of studies reported/total (%)	No. of studies reported as primary outcome/total (%)	No. of studies reported as secondary outcome/total (%)	No. of studies reported with definition/total (%)
TG	II. Pathological or pathophysiological	**3211**/3702 (86.7%)	**94**/3211 (2.9%)	12/3211 (0.4%)	**73**/3211 (2.3%)

TC	II. Pathological or pathophysiological	**3185**/3702 (86.0%)	**74**/3185 (2.3%)	**14**/3185 (0.4%)	**72**/3185 (2.3%)

Total effective rate	V. Response rate	**2780**/3702 (75.1%)	**18**/2780 (0.6%)	**6**/2780 (0.2%)	**2648**/2780 (95.3%)

HDL-C	II. Pathological or pathophysiological	**2709**/3702 (73.2%)	**63**/2709 (2.3%)	**17**/2709 (0.6%)	**63**/2709 (2.3%)

LDL-C	II. Pathological or pathophysiological	**2240**/3702 (60.5%)	**79**/2240 (3.5%)	**9**/2240 (0.4%)	**51**/2240 (2.3%)

Liver function	III. Adverse event or safety	**1199**/3702 (32.4%)	**1**/1199 (0.1%)	**6**/1199 (0.5%)	**10**/1199 (0.8%)

Renal function	III. Adverse event or safety	**1046**/3702 (28.3%)	**1**/1046 (0.1%)	**6**/1046 (0.6%)	**10**/1046 (1.0%)

Blood routine examination	III. Adverse event or safety	**958**/3702 (25.9%)	**1**/958 (0.1%)	**5**/958 (0.5%)	**9**/958 (0.9%)

Urine routine examination	III. Adverse event or safety	**873**/3702 (23.6%)	**1**/873 (0.1%)	**5**/873 (0.6%)	**7**/873 (0.8%)

ECG	II. Pathological or pathophysiological	565/3702 (15.3%)	0/565 (0.0%)	3/565 (0.5%)	7/565 (1.2%)

TG: total triglyceride; TC: total cholesterol; HDL-C: high-density lipoprotein cholesterol; LDL-C: low-density lipoprotein cholesterol; ECG: electrocardiogram.

**Table 3:** Subcategories of pathological or pathophysiological outcomes and frequency of outcomes reported in each subcategory.

No.	Outcome subcategories	No. of individual outcomes in each subcategory	Frequency of outcomes reported from this subcategory
Total		303	17,036
1	Blood lipid related outcomes	37	11,744
2	Blood glucose related outcomes	7	498
3	Steroid related outcomes	6	6
4	Protein related outcomes	43	845
5	Other lipid related outcomes	4	8
6	Carotid atherosclerotic plaque related outcomes	7	31
7	Heart function related outcomes	5	288
8	Hemorheology related outcomes	24	1,136
9	Cellular immune function related outcomes	5	8
10	Fluidity of cell membrane related outcomes	2	2
11	Inflammation related outcomes	7	118
12	Blood pressure related outcomes	2	363
13	Blood platelet function related outcomes	9	68
14	Blood coagulation function related outcomes	14	78
15	Enzymatic activity measurement outcomes	14	75
16	Hormone related outcomes	7	11
17	Insulin related outcomes	5	15
18	Imageological examination related outcomes	18	80
19	ECG related outcomes	2	622
20	Electrolyte related outcomes	8	99
21	Prostaglandin related outcomes	2	15
22	Stable no. of metabolites	1	1
23	Obesity related outcomes	7	15
24	Anemia related outcomes	4	4
25	Microcirculation related outcomes	10	12
26	Thrombotic test in vitro related outcomes	4	9
27	Blood gas analysis related outcomes	2	10
28	General items related outcomes	15	773
29	Physical examination related outcomes	5	16
30	Others	27	86

## Data Availability

The data used to support the findings of this study are available from the corresponding author upon request.

## Abbreviations

COS: Core outcome set
COMET: Core outcome measures in effectiveness trials
CM: Chinese medicine
HDL-C: High-density lipoprotein cholesterol
LDL-C: Low-density lipoprotein cholesterol
TC: Total cholesterol
TG: Total triglyceride
PRISMA: The preferred reporting items for systematic reviews and meta-analyses statement.

## Supplementary Materials

Supplementary Materials S1: search strategy. S2: outcome details.
