# Supplementary material for: Outcome Reporting Variability in Trials of Chinese Medicine for Hyperlipidemia: A Systematic Review for Developing a Core Outcome Set
Source: Evid Based Complement Alternat Med. 2021 Jun 10;2021:8822215. doi: 10.1155/2021/8822215 (PMC8211496; doi:10.1155/2021/8822215)
Supplement: Supplementary Materials — S1: search strategy. S2: outcome details. [file 8822215.f1.zip › 8822215.f1/Supplementary Materials S1 (search strategy) (2).doc]

| **Search strategy** | **Results** |
| --- | --- |
| **Database PubMed** |  |
| #1 hypercholesterolemia or hyperlipoproteinemias or hypertriglyceridemia or dyslipidemias or "Cholesterol, LDL" or "Cholesterol, HDL" or "Lipoproteins, HDL" or "Lipoproteins, LDL"  #2 hyperlipidaemia* or Hypercholesterolaemi* or hypertriglyceridaemia* or Hyperlipoproteinaemia*  #3 "blood lipids"[tiab] or "cholesterol"[tiab] or "triglyceride"[tiab] or “high density lipoprotein”[tiab] or “low density lipoprotein”[tiab] or "HDL"[tiab] or "LDL"[tiab]  #4 (#1 or #2 or #3) | 330095 |
| #5 “Traditional Chinese Medicine OR Chinese Traditional Medicine OR Chinese Herbal Drugs OR Chinese Drugs, Plant OR Medicine, Traditional OR Ethnopharmacology OR Ethnomedicine OR Ethnobotany OR Medicine, Kampo OR Kanpo OR TCM OR T.C.M. OR Medicine, Ayurvedic OR Alternative Medicine OR Complementary Medicine OR Phytotherapy OR Herbology OR Plants, Medicinal OR Plant Preparations OR Plant Extracts OR Plants, Medicine OR Materia Medica OR Single Prescription OR Herbs OR Chinese Medicine Herb OR Herbal Medicine”  #6 Acupuncture OR Meridians OR Electroacupuncture OR Moxibustion OR Auriculotherapy OR plum blossom OR acupressure OR ear acupuncture OR ear acupressure OR acupuncture, ear OR acupuncture therapy OR moxa OR laser acupuncture OR seven star needle OR acupuncture analgesia OR acupuncture points OR electro-acupuncture OR electro acupuncture OR TENS OR transcutaneous nerve stimulation OR transcutaneous electric nerve stimulation OR transcutaneous electrical nerve stimulation OR electro-stimulation OR electro stimulation OR pharmacopuncture OR point injection OR catgut embedding  #7 Tai Ji OR Tai chi OR Breathing exercises OR Qi gong OR Qigong OR Chi Kung OR Tuina OR anmo Tuina OR Chinese massage OR cupping OR guasha OR blood letting OR bloodletting OR diet therapy OR therapy, diet OR therapies, diet OR phlebotomy  #8 (#5 OR #6 OR #7) | 797312 |
| #9 “randomized controlled trial”[pt] OR “controlled clinical trial”[pt] OR “randomized”[tiab] OR “placebo”[tiab] OR “drug therapy”[sh] OR “randomly”[tiab] OR “trial”[tiab] OR “groups”[tiab]  #10 “Systematic” [sb]  #11 “cohort studies”[mesh] OR “case-control studies”[mesh] OR “comparative study”[pt] OR “risk factors”[mesh] OR “cohort”[tw] OR “compared”[tw] OR “groups”[tw] OR “case control”[tw] OR “multivariate”[tw] OR “case series”  #12 (#9 OR #10 OR #11) | 8562338 |
| #13 (#4 AND #8 AND #12) | 23352 |
| #14 Filters: Humans | 15077 |
| **Database EmBase** |  |
| #1 'hyperlipidemias'/exp OR 'hypertriglyceridemia'/exp OR 'hyperlipoproteinemias'/exp OR 'hypercholesterolemia'/exp OR 'dyslipidemias'/exp OR 'cholesterol, ldl'/exp OR 'cholesterol, hdl'/exp OR 'lipoproteins, hdl'/exp OR 'lipoproteins, ldl'/exp  #2 'hypercholesterolemia':ti OR 'hyperlipoproteinemias':ti OR 'hypertriglyceridemia':ti OR 'dyslipidemias':ti OR 'cholesterol, ldl':ti OR 'cholesterol, hdl':ti OR 'lipoproteins, hdl':ti OR 'lipoproteins, ldl':ti  #3 'hyperlipid?emia*':ti OR 'hypercholesterol?emi*':ti OR 'hypertriglycerid?emia*':ti OR 'hyperlipoprotein?emia*':ti  #4 'blood lipids':ti OR 'cholesterol':ti OR 'triglyceride':ti OR 'high density lipoprotein':ti OR 'low density lipoprotein':ti OR 'hdl':ti OR 'ldl':ti  #5 (#1 or #2 or #3 or #4) | 377075 |
| #6 'Chinese medicine' OR 'medicinal plant' OR 'Chinese drug' OR 'plant medicinal product' OR 'plant extract' OR 'herb' OR 'herbal medicine' OR 'material medica' OR 'traditional medicine' OR 'ethnopharmacology' OR 'ethnobotany' OR 'Kampo' OR 'Avuryeda' OR 'alternative medicine' OR 'phytotherapy'  #7 ‘acupuncture’ OR ‘electroacupuncture’ OR ‘moxibustion’ OR ‘ear acupuncture’ OR ‘plum blossom’ OR ‘acupressure’ OR ‘ear acupressure’ OR ‘acupuncture, ear’ OR ‘moxa’ OR ‘laser acupuncture’ OR ‘seven star needle’ OR ‘acupuncture analgesia’ OR ‘acupuncture points’ OR ‘electro-acupuncture’ OR ‘electro acupuncture’ OR ‘TENS’ OR ‘transcutaneous nerve stimulation’ OR ‘transcutaneous electric nerve stimulation’ OR ‘transcutaneous electrical nerve stimulation’ OR ‘electro-stimulation’ OR ‘electro stimulation’ OR ‘pharmacopuncture’ OR ‘point injection’ OR ‘catgut embedding’  #8 ‘Tai Chi’ OR ‘Tai Ji’ OR ‘Breathing exercise’ OR ‘Qi gong’ OR ‘Qigong’ OR ‘Tuina’ OR ‘anmo Tuina’ OR ‘Chinese massage’ OR ‘cupping’ OR ‘guasha’ OR ‘blood letting’ OR ‘bloodletting’ OR ‘Diet therapy’ OR ‘diet treatment’ OR ‘dietary therapy’ OR ‘dietary treatment’ OR ‘Phlebotomy’  #9 (#6 OR #7 OR #8) | 495463 |
| #10 ‘randomized controlled trial’/exp OR ‘controlled clinical trial’/exp OR ‘randomized’:ti,ab OR ‘placebo’:ti,ab OR ‘drug therapy’:lnk OR ‘randomly’:ti,ab OR ‘trial’:ti,ab OR ‘groups’:ti,ab  #11 'meta analysis (topic)' OR 'systematic review (topic)' OR review.it  #12 ‘clinical article’/exp OR ‘controlled study’/exp OR ‘major clinical study’/exp OR ‘prospective study’/exp OR ‘cohort analysis’/exp OR ‘cohort’:ti,ab OR ‘groups’:ti,ab OR ‘case control’:ti,ab OR ‘multivariate’:ti,ab OR ‘case series’:ti,ab  #13 (#10 OR #11 OR #12)  #14 (#5 AND #9 AND #13)  #15 ([article]/lim OR [article in press]/lim OR [conference paper]/lim OR [conference review]/lim OR [review]/lim) | 12018 |
| **Database Cochrane Library** |  |
| #1 MeSH descriptor: [Hyperlipidemias] explode all trees  #2 MeSH descriptor: [Hypertriglyceridemia] explode all trees  #3 MeSH descriptor: [Hyperlipoproteinemias] explode all trees  #4 MeSH descriptor: [Hypercholesterolemia] explode all trees  #5 MeSH descriptor: [Dyslipidemias] explode all trees  #6 MeSH descriptor: [Cholesterol, LDL] explode all trees  #7 MeSH descriptor: [Cholesterol, HDL] explode all trees  #8 MeSH descriptor: [Lipoproteins, HDL] explode all trees  #9 MeSH descriptor: [Lipoproteins, LDL] explode all trees  #10 (#1 or #2 or #3 or #4 or #5 or #6 or #7 or #8 or #9) | 10145 |
| #11 hypercholesterolemia or hyperlipoproteinemias or hypertriglyceridemia or dyslipidemias:ti,ab,kw (Word variations have been searched)  #12 hyperlipidaemia* or Hypercholesterolaemi* or hypertriglyceridaemia* or Hyperlipoproteinaemia*:ti,ab,kw (Word variations have been searched)  #13 blood lipids or cholesterol or triglyceride or "high density lipoprotein" or "low density lipoprotein" or HDL or LDL  #14 (#10 or #11 or #12 or #13 ) | 36430 |
| #15 Traditional Chinese Medicine or Chinese Traditional Medicine or Chinese Herbal Drugs or Chinese Drugs, Plant or Medicine, Traditional or Ethnopharmacology or Ethnomedicine or Ethnobotany or Medicine, Kampo or Kanpo or TCM or T.C.M. or Medicine, Ayurvedic or Alternative Medicine or Complementary Medicine or Phytotherapy or Herbology or Plants, Medicinal or Plant Preparations or Plant Extracts or Plants, Medicine or Materia Medica or Single Prescription or Herbs or Chinese Medicine Herb or Herbal Medicine:ti,ab,kw (Word variations have been searched)  #16 Acupuncture or Meridians or Electroacupuncture or Moxibustion or Auriculotherapy or plum blossom or acupressure or ear acupuncture or ear acupressure or acupuncture, ear or acupuncture therapy or moxa or laser acupuncture or seven star needle or acupuncture analgesia or acupuncture points or electro-acupuncture or electro acupuncture or TENS or transcutaneous nerve stimulation or transcutaneous electric nerve stimulation or transcutaneous electrical nerve stimulation or electro-stimulation or electro stimulation or pharmacopuncture or point injection or catgut embedding:ti,ab,kw (Word variations have been searched)  #17 Tai Ji or Tai chi or Breathing exercises or Qi gong or Qigong or Chi Kung or Tuina or anmo Tuina or Chinese massage or cupping or guasha or blood letting or bloodletting or diet therapy or therapy, diet or therapies, diet or phlebotomy:ti,ab,kw (Word variations have been searched)  #18 (#15 or #16 or #17) | 74098 |
| #19 (#14 and #18) | 5500 |
| **Database CNKI** |  |
| SU=("高脂血症"+"高胆固醇血症"+"高甘油三脂血症"+"高脂蛋白血症"+"脂代谢异常"+"血脂异常"+"血脂过多") and SU=("中医"+"中西医"+"中医疗法"+"辨病论治"+"辨证"+"辨证论治"+"辨症施治"+"汉方"+"祖国医学"+"传统医学"+"传统治疗"+"替代医学"+"替代治疗"+"中国传统医学"+"民族医药"+"草药"+"中草药"+"中药"+"中药疗法"+"中西药"+"传统医药"+"中成药"+"植物药"+"中医治法"+"熏洗"+"药浴"+"沐足"+"足浴"+"灌肠"+"熨法"+"药熨"+"熨药"+"热熨"+"热敷"+"敷脐"+"药枕"+"药烘") and SU=("临床观察"+"临床评估"+"临床试验"+"临床效果"+"临床研究"+"疗效"+"评价研究"+"前瞻性"+"随访"+"对比研究"+"多中心"+"随机"+"对照"+"病例报告"+"病例研究"+"病例分析"+"病例报道"+"系统评价"+"meta"+"荟萃分析"+"系统分析"+"综述") | 4239 |
| SU=("高脂血症"+"高胆固醇血症"+"高甘油三脂血症"+"高脂蛋白血症"+"脂代谢异常"+"血脂异常"+"血脂过多") and SU=("针刺"+"灸"+"针法"+"刺法"+"体针"+"腹针"+"温针"+"火针"+"电针"+"电磁针"+"梅花针"+"水针"+"穴位注射"+"经络注射"+"穴位按压"+"穴位按摩"+"指压穴位"+"耳压"+"耳针"+"耳穴"+"耳豆"+"埋藏疗法"+"埋线"+"外治"+"推拿"+"按摩"+"拔罐"+"药罐"+"推罐"+"闪罐"+"电罐"+"火罐"+"砭石"+"砭术"+"砭法"+"刮痧"+"刮搓"+"挑治"+"割治"+"发泡"+"导引"+"吐纳"+"气功"+"太极"+"八段锦"+"刺络"+"刺血"+"放血"+"三棱针") and SU=("临床观察"+"临床评估"+"临床试验"+"临床效果"+"临床研究"+"疗效"+"评价研究"+"前瞻性"+"随访"+"对比研究"+"多中心"+"随机"+"对照"+"病例报告"+"病例研究"+"病例分析"+"病例报道"+"系统评价"+"meta"+"荟萃分析"+"系统分析"+"综述") | 744 |
| The sum of the parts above | 4983 |
| **Database CBM** |  |
| #1(高脂血症 or 高胆固醇血症 or 高甘油三酯血症 or 高脂蛋白血症 or 血脂过多or脂代谢异常 or 血脂异常) AND (人类[特征词]) |  |
| #2中医 OR 中西医 OR 辨病论治 OR 辨证 OR 辨症施治 OR 汉方 OR 祖国医学 OR 传统医学 OR 传统治疗 OR 替代医学 OR 替代治疗 OR 民族医药 OR 草药 OR 中草药 OR 中药 OR 中西药 OR 传统医药 OR 中成药 OR 植物药 OR 中医治法 OR 熏洗 OR 药浴 OR 沐足 OR 足浴 OR 灌肠 OR 熨法 OR 药熨 OR 熨药 OR 热熨 OR 热敷 OR 敷脐 OR 药枕 OR 药烘 OR 针刺 OR 灸 OR 针法 OR 刺法 OR 体针 OR 腹针OR 温针 OR 火针 OR 电针 OR 电磁针 OR 梅花针 OR 水针 OR 穴位注射 OR 经络注射 OR 穴位按压 OR 穴位按摩 OR 指压穴位 OR 耳压 OR 耳针OR 耳穴 OR 耳豆 OR 埋藏疗法 OR 埋线 OR 外治 OR 推拿OR 按摩 OR 拔罐 OR 药罐 OR 推罐 OR 闪罐 OR 电罐 OR 火罐 OR 砭石 OR 砭术 OR 砭法 OR 刮痧 OR 刮搓 OR 挑治 OR 割治 OR 发泡 OR 导引 OR 吐纳 OR 气功 OR 太极 OR 八段锦 OR 刺络 OR 刺血 OR 放血 OR 三棱针 |  |
| #3临床观察 OR 临床评估 OR 临床试验 OR 临床效果 OR 临床研究 OR 疗效 OR 评价研究 OR 前瞻性 OR 随访 OR 对比研究 OR 多中心 OR 随机 OR 对照 OR 病例报告 OR 病例研究 OR 病例分析 OR 病例报道 OR系统评价 OR meta 荟萃分析 OR 系统分析 OR 综述 |  |
| #4(#1 AND #2 AND #3) | 8032 |
| **Database Wanfang** |  |
| 题名或关键词:(脂代谢异常+血脂异常+高脂血症+高胆固醇血症+高甘油三酯血症+高脂蛋白血症+血脂过多) and 题名或关键词:(临床观察+临床评估+临床试验+临床效果+临床研究+疗效+评价研究+前瞻性+随访+对比研究+多中心+随机+对照+病例报告+病例研究+病例分析+病例报道+系统评价+meta+荟萃分析+系统分析+综述) | 6295 |
